# Genetic profiles and phenotypic patterns in Taiwanese Phalaenopsis orchids: a two-step phenotype and genotype strategy using modified genetic distance algorithms

**DOI:** 10.3389/fpls.2024.1416886

**Published:** 2024-09-11

**Authors:** Ya-Syuan Lai, Shu-Yun Chen, Yan-Jeng Wu, Wen-Huei Chen, Hong-Hwa Chen, Yung-Yu Lin, Te-Cheng Lin, Te-Ju Lin, Chung-Feng Kao

**Affiliations:** ^1^ Department of Agronomy, College of Agriculture and Natural Resources, National Chung Hsing University, Taichung, Taiwan; ^2^ Orchid Research and Development Center, National Cheng Kung University, Tainan, Taiwan; ^3^ Department of Life Sciences, National Cheng Kung University, Tainan, Taiwan; ^4^ Research and Development Department, Brother Orchid Nursery Co., Ltd., Taichung, Taiwan; ^5^ Research and Development Department, Wonderorchids Co., Ltd., Taichung, Taiwan

**Keywords:** 2-step P+G strategy, core collection, diversity, germplasm, modified genetic distance, Phalaenopsis orchids

## Abstract

This study establishes the first core collection (CC) for Taiwanese Phalaenopsis orchids to preserve genetic diversity and key traits essential for breeding and research, thereby enhancing breeding efficiency without the need for a large maintained parent population. We examined 207 commercial orchid cultivars from ten nurseries, characterized by two phenotypes and genotypic data from eight simple sequence repeat markers. Multiple imputation was applied to estimate missing phenotypes, minimizing potential uncertainties and ensuring the reliability of population structure analysis. Weighted *k*-means clustering identified seven distinct clusters, highlighting substantial genetic diversity. We proposed a two-step phenotype and genotype strategy and modified genetic distance algorithms to effectively preserve both phenotypic and genetic diversity while retaining key features. Consequently, 22 core accessions were selected, distributed across seven clusters, and representing the orchid germplasm collection. Our evaluation revealed significant diversity preservation, particularly in distinct characteristics and rare features, outperforming other methodologies. Pedigree background analysis further confirmed the representativeness of the CC in maintaining diverse genetic materials. We emphasized the importance of evaluating the CC by detailing the criteria and statistical analyses used to ensure the quality, representativeness, and effectiveness of the selected accessions. This study contributes to orchid breeding, conservation efforts, and sustainable agricultural practices by providing a valuable and comprehensive resource. In conclusion, our research establishes a groundbreaking CC, offering insights into the genetic landscape of Taiwanese Phalaenopsis orchids and highlighting potential advancements in breeding commercially desirable varieties.

## Introduction

Orchids are among the most significant ornamental plants worldwide, holding substantial global export value (Hsu et al., 2022). Within the vast angiosperm family Orchidaceae, which comprises 27,315 species, the *Phalaenopsis* genus stands out with around 92 species (Bidarnamani et al., 2020). This genus is particularly important due to its extensive cultivation, as evidenced by the approximately 35,000 officially registered orchid hybrids by the Royal Horticultural Society (Hsu et al., 2022). In Taiwan, *Phalaenopsis* orchids are economically crucial, contributing an annual export value of 140 million US dollars, which represents 75% of the total orchid export value (https://www.coa.gov.tw/). This underscores their significant economic impact in the region.

For orchid breeders, developing commercially valuable orchids involves creating thousands of hybrids from hundreds of parent plants, necessitating a diverse genetic background and considerable time (Tang and Chen, 2007). The high labor costs and the need for extensive genetic diversity further complicate breeding programs. Current methods lack efficiency in reducing redundancy within the parent populations, leading to unsustainable practices. Although core collections (CCs) have proven effective in other plant species for preserving genetic diversity and reducing maintenance efforts, no such CC has been established for Phalaenopsis orchids using morphological or molecular marker techniques. This study addresses the urgent need for a sustainable and efficient approach to managing Phalaenopsis orchid breeding programs by developing a core collection that accurately represents the genetic diversity of the species.

A notable advancement in conserving genetic materials was the introduction of CCs, which involves selecting a representative subset that encapsulates the genetic diversity of the original collection (Kao et al., 2021). CCs are invaluable for facilitating experimental research aimed at utilizing genetic materials in various scientific endeavors (Diwan et al., 1995). The development of CCs has employed various methodologies, including the use of morphological and geological data, as seen in studies on peanuts (Wann et al., 2020), quinoa (Craine et al., 2023), and soybean (Kao et al., 2021).

Molecular markers, particularly simple sequence repeats (SSR) markers, have been widely used in genetic research to analyze DNA-level variations across species and populations. SSR markers have proven effective in multiple studies. For instance, SSR markers were used in rice to examine 2,260 varieties, resulting in the selection of 19 cultivars for a core collection (Zhang et al., 2011). In strawberries, SSR markers facilitated the selection of 19 cultivars from a pool of 119 for a CC (Wada et al., 2017). Additionally, a CC comprising 32 strains of winter mushroom was formed from 81 different F. veltuipes strains using SSR markers (Liu et al., 2018). Integrating morphological traits with SSR markers has successfully established CCs for various species, such as *Camellia oleifera* (Zhu et al., 2022) and *Perilla frutescens* (Sa et al., 2021), selecting 25 and 44 accessions from 167 and 400 germplasms, respectively. These examples highlight the efficacy of SSR markers for developing CCs, demonstrating their potential when combined with morphological data.

Despite the extensive use of SSR markers in developing CCs for various species, no CC has been established for *Phalaenophsis* orchids using either morphological or molecular marker techniques. This gap presents an opportunity to apply these proven methodologies to *Phalaenophsis*, potentially enhancing genetic diversity and sustainability in its cultivation.

In practical terms, morphological traits such as flower diameter and plant height are critical for orchid breeders in Taiwan when assessing the market potential of cultivars. Orchid flowers are categorized into three ranks: “large flower” (exceeding 10 cm), “medium flower” (7 to 9 cm), and “small flower” (below 6 cm) (Yang and Hu, 2016). Additionally, plant height is classified as “short,” “medium,” and “long”. While SSR markers have been used for orchid cultivar identification based on genetic identity (Chung et al., 2017; Han, 2005), they have not yet been employed in establishing a CC for orchids.

Researchers have developed various sampling methods for establishing CCs using both morphological and molecular data. These methods include the proportion strategy (P strategy), the constant strategy (C strategy), and the logarithmic strategy (L strategy) (Brown, 1989). Among these, the maximization (M) strategy is the most prevalent, aiming to maximize allele diversity while retaining genetic richness with minimal redundancy. Algorithms based on the M strategy, such as MSTRAT (Gouesnard et al., 2001), Powercore (Kim et al., 2007), and Corehunte (Thachuk et al., 2009), have been developed to enhance genetic diversity in CCs.

**Figure 1:**
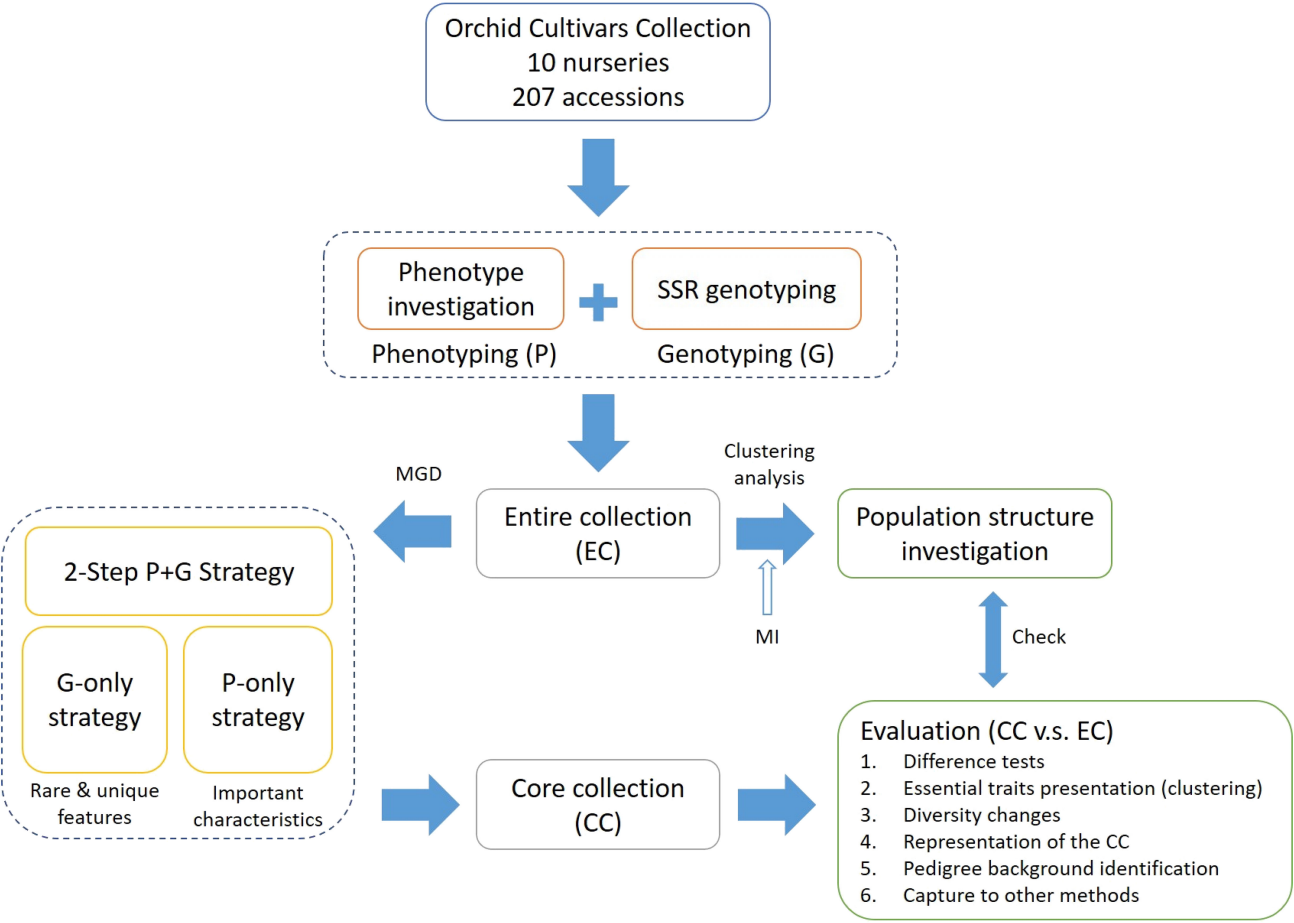
Methodological framework for establishing a core collection of Taiwanese Phalaenopsis orchids.

In this study, we aimed to develop an optimal CC from 207 orchid varieties collected in Taiwan by introducing a modified genetic distance (MGD) algorithm. This algorithm is designed to manage the variability in phenotypic and genotypic data for orchid accessions. To construct a CC that accurately represents Phalaenopsis orchid germplasms, we devised a two-step phenotype and genotype strategy, denoted as ‘P+G strategy’ focused on capturing distinct and heritable characteristics, with an emphasis on unique and rare traits. The effectiveness and validation of the core accessions were assessed by comparing the phenotypic and genotypic diversity between the CC and the entire germplasm collection, as well as through pedigree background analysis. Additionally, to evaluate the robustness of the CC, comparisons were made with other selection methods. The CC developed through this method aims to address challenges such as maintenance costs and promote sustainable practices, aiding breeders in managing their breeding programs more efficiently in Taiwan. For a comprehensive overview of the study’s framework, please refer to **Figure 1**.

## Materials and methods

### Orchid cultivars collection

A total of 203 *Phalaenopsis* orchid cultivars were collected, consisting of 197 commercial cultivars and 6 native species. These germplasms were sourced from 10 distinct orchid nurseries located in Taichung, Changhua, Tainan, and Pingtung in Taiwan (please refer to **Supplementary Table 1**). Orchid nurseries were selected based on several key criteria, including geographical diversity, genetic variability, management practices, and availability of data. These criteria were meticulously applied to ensure that the selected orchid nurseries would provide a robust and representative sample for the study. To further enhance genetic diversity coverage, we also included 4 orchid species of unknown origin.

### Morphological data collection

To ensure accurate identification of orchid phenotypes, two key flower-related traits were measured: plant height and flower diameter. Data collection occurred after the flower forcing by orchid nurseries during the peak flowering season to capture phenotypic characteristics at their most representative stage. Plant height (cm) was measured from the base of the plant at the soil surface to the apex, including any flower buds or fully developed flowers. This measurement was standardized to take place when the plants were in full bloom, ensuring consistency across samples. Measurements were recorded using a calibrated tape measure on three randomly selected plants per accession within each greenhouse, ensuring that intra-nursery variability was accounted for. Flower diameter (cm) was measured by the maximum horizontal span of the fully expanded flower using precision calipers. This measurement was conducted simultaneously with the plant height measurement for each selected plant to minimize environmental influence. The calipers were chosen for their accuracy in capturing small variations in flower size. Data collection was performed by a trained team of experts to maintain consistency in measurement techniques across different nurseries. Each measurement was repeated three times for each plant to ensure reliability, with the average of the three readings being recorded as the final value. This approach minimized potential biases and ensured that the collected data accurately reflected the morphological diversity present in the Phalaenopsis germplasm.

### DNA extraction

The newly emerged leaf from each cultivar was carefully excised and used for DNA extraction. In this study, we employed a modified version of the plant CTAB DNA extraction protocol (Porebski et al., 1997). The quality of all DNA samples was assessed using a Nanodrop Lite spectrophotometer (Thermo Scientific) to determine the 260/280 ratio. Additionally, a 1% agarose gel electrophoresis at 100V for 30 minutes was conducted to further verify DNA integrity. Following these aforementioned quality control steps, the extracted DNA samples were stored at -20°C for future analysis.

### SSR genotyping

DNA from 207 cultivars was quantified and diluted to a concentration of 20 ng/*μ*L, as determined by nanodrop measurements. Subsequently, 2 *μ*L of DNA (20 ng/*μ*L) was combined with 2 *μ*L of 5 *μ*M forward and reverse SSR markers for the PCR process. We applied 8 SSR markers (Lee et al., 2021), as these markers displayed strong polymorphism among orchid cultivars after analysis. The sequences of SSR primers are provided in **Supplementary Table 2**. The PCR program was conducted as follows: initial denaturation for 5 minutes at 94°C, followed by 35 cycles of denaturation (15 seconds at 94°C), annealing (15 seconds at 60°C), and extension (30 seconds at 72°C), and finally, a 5-minute extension step at 72°C. After PCR, we utilized a 5% acrylamide gel to distinguish the product sizes from each SSR marker.

### Multiple imputation of missing data in correlated phenotypes

**Figure 2:**
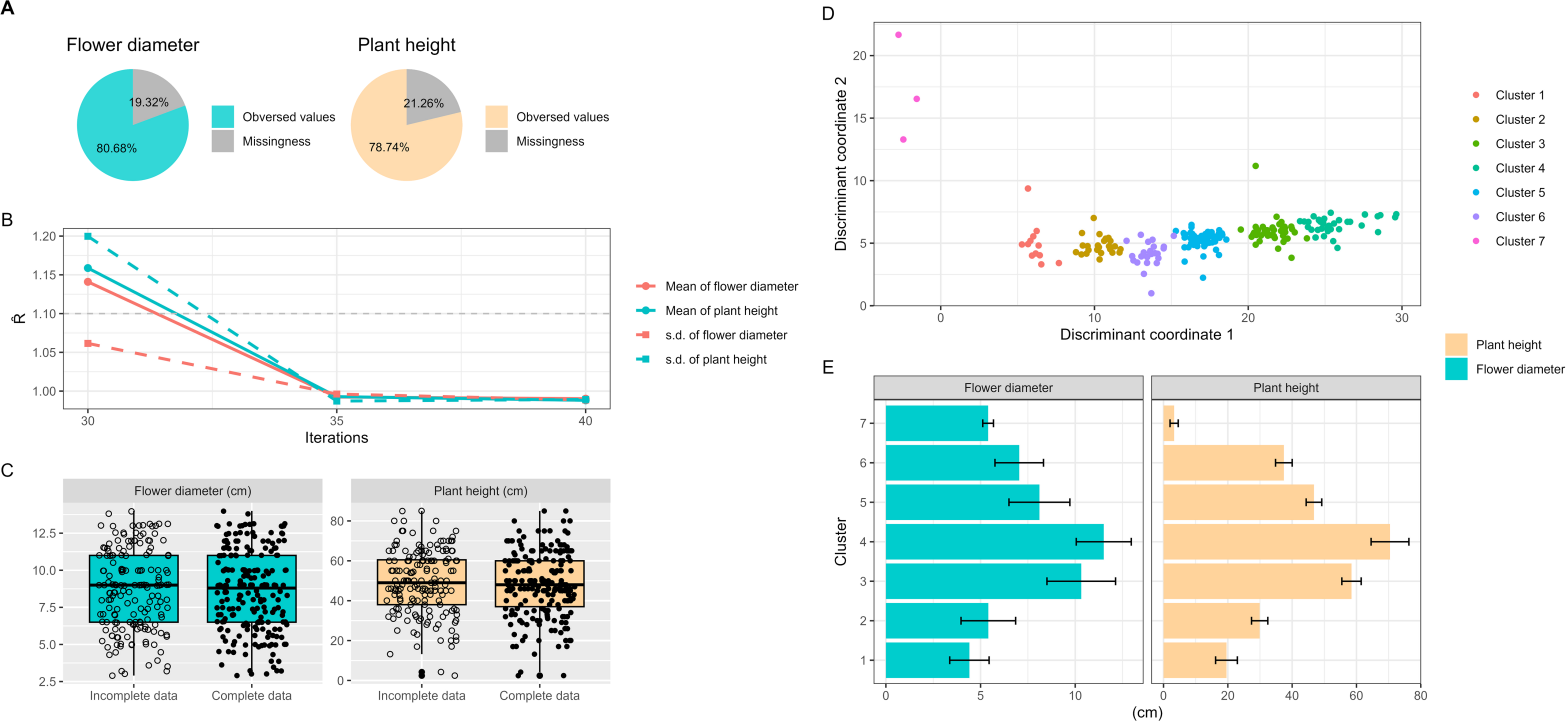
Analysis of missing phenotypes, multiple imputation and cluster analysis in Taiwanese Phalaenopsis orchids germplasm. **(A)** Presentation of missing phenotypic trait rate. **(B)** Estimation of convergence for imputed phenotypes using multiple imputation algorithm. Solid and dashed line indicate the mean and standard deviation of phenotypes, respectively. Red and cyan colors represent plant height and flower diameter, respectively. Convergence is considered achieved when the $\hat{\text{R}}$ statistic for each trait’s mean and standard deviation falls within the 1.0 ± 0.1 convergence threshold. **(C)** Illustration of incomplete data (raw phenotypes) and complete data distribution (raw plus imputed phenotypes). **(D)** Implementation of weighted *k*-means clustering on mixed phenotypic and genotypic data. The 207 Taiwanese Phalaenopsis orchids germplasm are classified into seven clusters. Solid and cross circles represent entire collection (EC) and core collection (CC), respectively, selected using the two-step ‘P+G strategy’ and the modified genetic distance algorithm. **(E)** Display of central tendency and dispersion of phenotypic traits within different clusters. The orange bar and cyan circle represent plant height and flower diameter, respectively.

MI is a statistical algorithm used to deal with missing data, especially in the context of correlated phenotypic data. The $\hat{R}$ statistic, computed to assess the coverage of iterations, is expected to be less than 1.1 (Su et al., 2011). The MI procedures were conducted using the *mi* package in R (ver. 4.1.2) (R Core Team, 2013). We used chained equations in multiple imputation to estimate the missing phenotypes in correlated traits over 35-40 iterations. To ensured successful convergence by verifying that the values of the $\hat{R}$ statistic for the mean and standard deviation of each trait were within the convergence threshold of 1.0 ± 0.1 (**Figure 2B**).

### Weighted *k*-means clustering

To investigate the population structure of orchid germplasm accessions, we applied weighted *k*-means clustering to analyze the mixed-type phenotypic and genotypic data of our germplasm collection. The determination of the optimal number of clusters was based on specific criteria. We aimed for clusters that exhibited high genetic diversity, often characterized by a Shannon–Weaver diversity and Nei’s diversity index exceeding 90% and 80%, respectively. Additionally, we sought clusters that explained a large portion of the variance, typically greater than 75%. The weighted *k*-means clustering algorithm was implemented using the *kamila* and *fpc* packages in the R programming language (ver. 4.1.2) (Foss and Markatou, 2018; R Core Team, 2013), allowing us to conduct this analysis effectively and efficiently.

### Dissimilarity metric utilizing a modified genetic distance

We introduced the MGD algorithm to quantify dissimilarity, or genetic distance, between pairs of accessions. This dissimilarity metric is capable of handling complex mixed-type data, including both quantitative and qualitative information, such as phenotypic and genotypic data. For quantitative data, we utilized the Euclidean distance, denoted as $d_{Eul}^{2}$ , to quantify dissimilarity among accessions in a multidimensional space. It is defined as


$$\sum_{j\in C}\sum_{i\neq i^{*}}{\left(x_{ij}-x_{i^{*}j}\right)}^{2}$$

Here, C represents the quantitative trait set, and $x_{ij}$ represents the phenotypic trait value of the $i^{th}$ accession in the $j^{th}$ trait. For qualitative data, we employed the inverse occurrence frequency (IOF) measure, denoted as $d_{IOF}^{2}$ , which assigns higher weight to mismatched characteristics among accessions, especially for less frequent values. It is defined as


$$\sum_{j\in Q}\sum_{i\neq i^{*}}{\left(logf_{ij}\cdot logf_{i^{*}j}\right)}^{2}$$

where Q and $f_{ij}$ represents the qualitative-trait set and the frequency of the $i^{th}$ accession in the $j^{th}$ trait, respectively. Hence, the MGD can be expressed as


$$d_{MGD}^{2}=\{\begin{array}{cc} 0, & \mathrm{if}\text{ the levels of a traits are the same between }i\;\text{and}\;i^{*}\\ d_{Eul}^{2}+d_{IOF}^{2}, & \text{if the levels of a trait are different between}\;i\;\text{and}\;i^{*} \end{array}$$

and this dissimilarity metric is employed to effectively capture unique and distinct characteristics within the intricate structure of combined phenotypic and genotypic data.

### Establishing a CC: a two-step ‘P+G strategy’

To select a CC with important characteristics from the orchid germplasm accessions, we developed a two-step ‘P+G strategy’ to capture essential characteristics and features within the intricate and diverse traits and genetic diversity of orchid germplasm. This approach involves using phenotypic data (‘P-only strategy’) to uncover rare and unique features and then leveraging genotypic data (‘G-only strategy’) to identify important characteristics. Unlike the traditional P+G strategy, our pipeline captures key features while preserving maximum germplasm diversity. The process for selecting core accessions includes these steps: (1) Missing data imputation: Missing phenotypes were generated using multiple imputation. (2) Germplasm structure exploration: We employed the weighted *k*-means clustering method to uncover the architectural structure of orchid germplasm. (3) Dissimilarity estimation: Genetic distances among accessions were assessed using the MGD algorithm. (4) CC construction: Core accessions were selected based on the MGD metric through the 2-step ‘P+G strategy’. (5) CC evaluation: We compared and evaluated differences in phenotypes, genetic diversity, changes in central tendency and dispersion of phenotypes, and pedigree background of orchid cultivars between the CC and the entire collection (EC). By meeting these conditions, a CC of germplasm can serve as a valuable resource for agricultural research, breeding programs, and conservation effort. A detailed pipeline for constructing the CC is illustrated in **Figure 1**.

**Figure 3:**
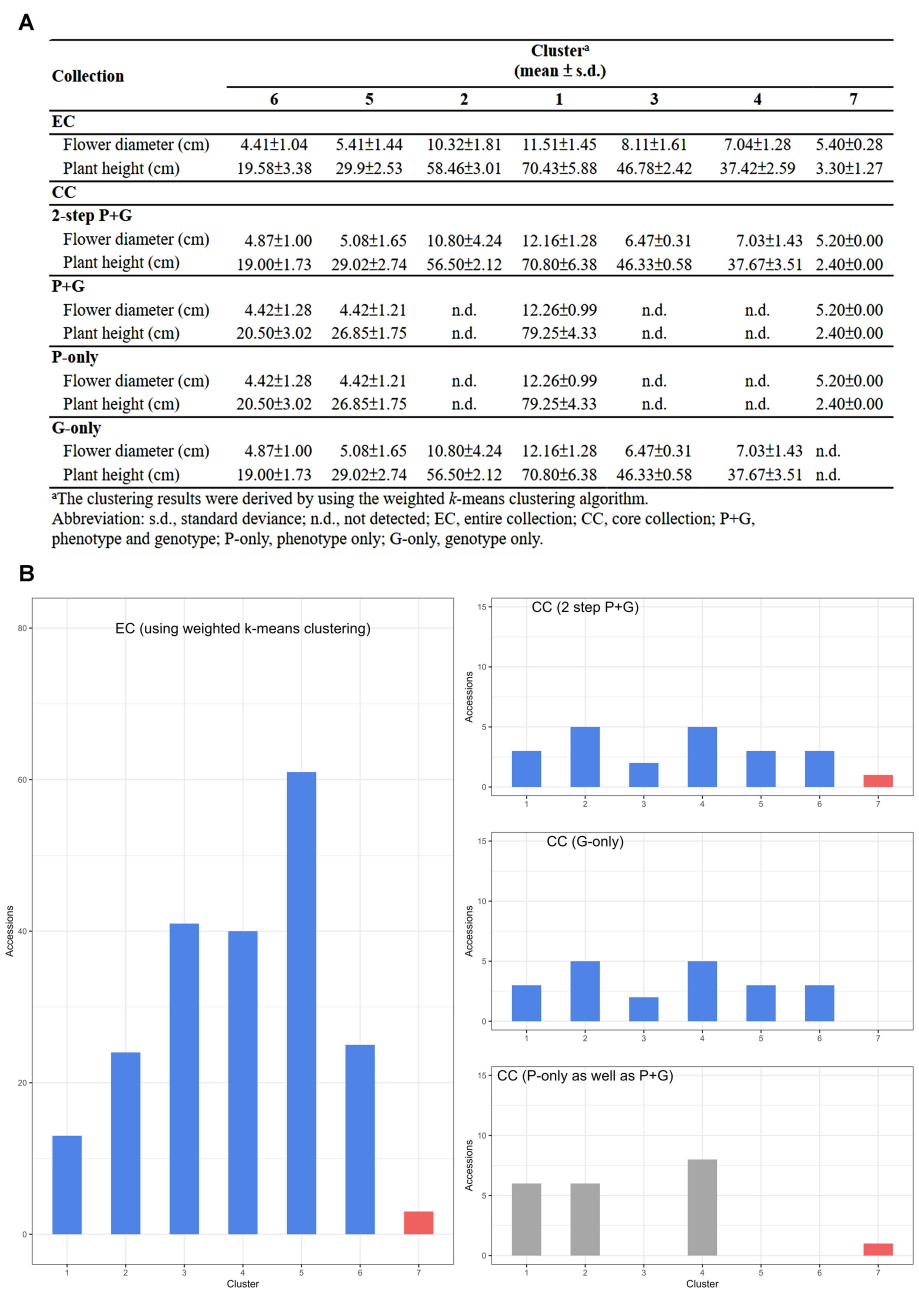
Effectiveness of the two-step ‘P+G strategy’ in CC selecting. **(A)** Depiction of central tendency and dispersion for the CC under different data entry strategies during CC selection. **(B)** Distribution of EC and CC across different strategies in seven different clusters. The upper panel displays the 2-step ‘P+G strategy’ results, revealing seven clusters. The middle panel shows the ‘G-only strategy’ outcomes, identifying six clusters and omitting the 7^th^ with rare characteristics. The results from both the ‘P-only strategy’ and traditional ‘P+G strategy’ are the same and showed in the lower panel, capturing noticeable rare features while missing other significant traits. Blue and grey bars indicate distinct and significant traits, respectively, with the red bar denoting rare characteristics in phenotypic traits.

We implemented the ‘P-only strategy’ to identify rare characteristics, specifically the smallest flowers (see **Figure 3B**, bottom right, cluster 7). Subsequently, we applied the ‘G-only strategy’ to identify other important characteristics (see **Figure 3B**, middle right, clusters 1-6).

### Evaluating the CC

The evaluation of the CC is crucial for its utility in various research domains. A high-quality CC of germplasm must meet several essential criteria, including a sufficient number of accessions (representative of the entire germplasm collection), high genetic diversity (to preserve genetic and phenotypic characterization), and minimal redundancy (for ease of management). These conditions are vital to ensure the effectiveness, reliability, and representativeness of the CC. By fulfilling these criteria, a CC of germplasm can become a valuable resource for agricultural research, breeding programs, and conservation efforts.

To comprehensively evaluate the CC, several statistical analyses were employed. Variations in central tendency and dispersion of phenotypic traits between the CC and the EC were examined. Initially, Levene’s test (Levene, 1960) with 1,000 iterations of bootstrap was performed to assess variance homogeneity. Subsequently, Student’s *t*-test (for equal variances) and Welch’s *t*-test (for unequal variances) were conducted to confirm the significance of the mean differences between the EC and the CC. Additionally, to gauge the representativeness of the CC concerning various phenotypic traits compared to the EC, metrics such as mean different percentage (MD%) (Hu et al., 2000), variance difference percentage (VD%), coincidence rate (CR%), and variable rate (VR%) were calculated. The formulas used for these metrics are as follows:


$$\text{MD\%}=\;\frac{\text{Frequency of significant mean difference}}{\text{Total number of comparisons}}\times 100\%$$

$$\text{VD\%}=\;\frac{1}{m}\sum_{i=1}^{m}\frac{\left|V_{e}-V_{c}\right|}{V_{c}}\times 100\%$$

$$\text{CR\%}=\;\frac{1}{m}\sum_{i=1}^{m}\frac{R_{c}}{R_{e}}\times 100\%$$

$$\text{VR\%}=\;\frac{1}{m}\sum_{i=1}^{m}\frac{CV_{c}}{CV_{e}}\times 100\%$$

where the symbols *V*, *R*, *CV*, and *m* represent variance, range, coefficient of variation, and the number of traits, respectively.

In addition to these analyses, phenotypic diversity within the CC was assessed using the Shannon diversity index (Shannon, 1948) and Nei’s diversity index (Nei, 1973) to evaluate evenness and richness in orchid abundance, respectively. The metrics are displayed as follows:


$$H^{\prime }=-\frac{\sum_{i=1}^{S}p_{i}\text{ln}\left(p_{i}\right)}{\text{ln}\left(S\right)}$$

$$Nei=1-\sum_{i=1}^{S}p_{i}^{2}$$

Here, $p_{i}$ represents the proportion of accessions within the *i* ^th^ group out of all germplasm data; S represents the number of clusters.

To assess the information content of genetic diversity among orchid genotypes for individual SSR primers, we calculated two quantitative measures, namely, polymorphic information content (PIC) (Roldan-Ruiz et al., 2000; De Riek et al., 2001), expected heterozygosity (Weir and Cockerham, 1968) and fixation index (F_ST_) (Nei, 1973). The PIC value, ranging between 0 and 1, serves as an indicator of the presence and absence of bands arising from polymorphism or variability observed among distinct alleles at specific microsatellite loci within the orchid population. Within our study, we employed SSR markers as dominant markers due to their efficacy in discernible band patterns on gel images following amplification across various heterozygous orchid individuals. The metric is depicted as follows:


$$PIC=\frac{1}{m}\sum_{i=0}^{m}\left\{1-\left(q_{i}^{2}+r_{i}^{2}\right)\right\}$$

where $q_{i}$ and $r_{i}$ represents the frequency of present and absent bands from each allele within the *i* ^th^ SSR allele for each primer, respectively; m represents total number of SSR alleles in the primer.

Expected heterozygosity, estimated according to Hardy-Weinberg equilibrium, is used to measure the diversity of the orchid population by assessing the expected value of heterozygosity within the population. The metric is represented as follows:


$$H_{exp}=1-\sum_{i=0}^{m}p_{i}^{2}$$

Where m represents total number of SSR alleles in the primer; $p_{i}$ represents the frequency of the *i* ^th^ SSR allele in the primer

Fixation index (F_ST_) serves as an informative measure of genetic diversity within the orchid population, offering valuable insights into the abundance and distribution of alleles. This quantitative metric spans a range from 0 to 1, wherein higher values signify heightened genetic diversity or heterozygosity at the particular SSR marker under examination. The metric is represented as follows:


$$H_{S}=\frac{1}{m}\sum_{i=1}^{m}\left(1-\sum_{j=1}^{n}p_{j}^{2}\right)$$

$$H_{T}=1\sum {\bar{P}}_{j}^{2}$$

$$F_{ST}=\frac{H_{T}-H_{S}}{H_{T}}$$

*H*_S_ is calculated for *n* alleles with a frequency of $p_{j}$ , where ${\bar{P}}_{j}$ represents the mean allele frequency of allele *j* across all subpopulations. *m* represents the number of subpopulations.

Moreover, pedigree background identification of orchid cultivars was carried out. Out of the 203 examined cultivars, 79 could be traced back to their pedigree as they were registered with The Royal Horticultural Society (https://www.rhs.org.uk/). The genetic background of these cultivars was documented based on the contributions of 17 distinct native *Phalaenopsis* species, utilizing the OrchidWiz Orchid Database Software (https://www.orchidwiz.com/). Subsequently, the correlation between the known pedigree of these 79 cultivars and 12 cultivars within the CC, based on 17 native parental species, was estimated using the R software.

## Results

### Characterization and relation analysis among orchid nurseries

**Figure 4:**
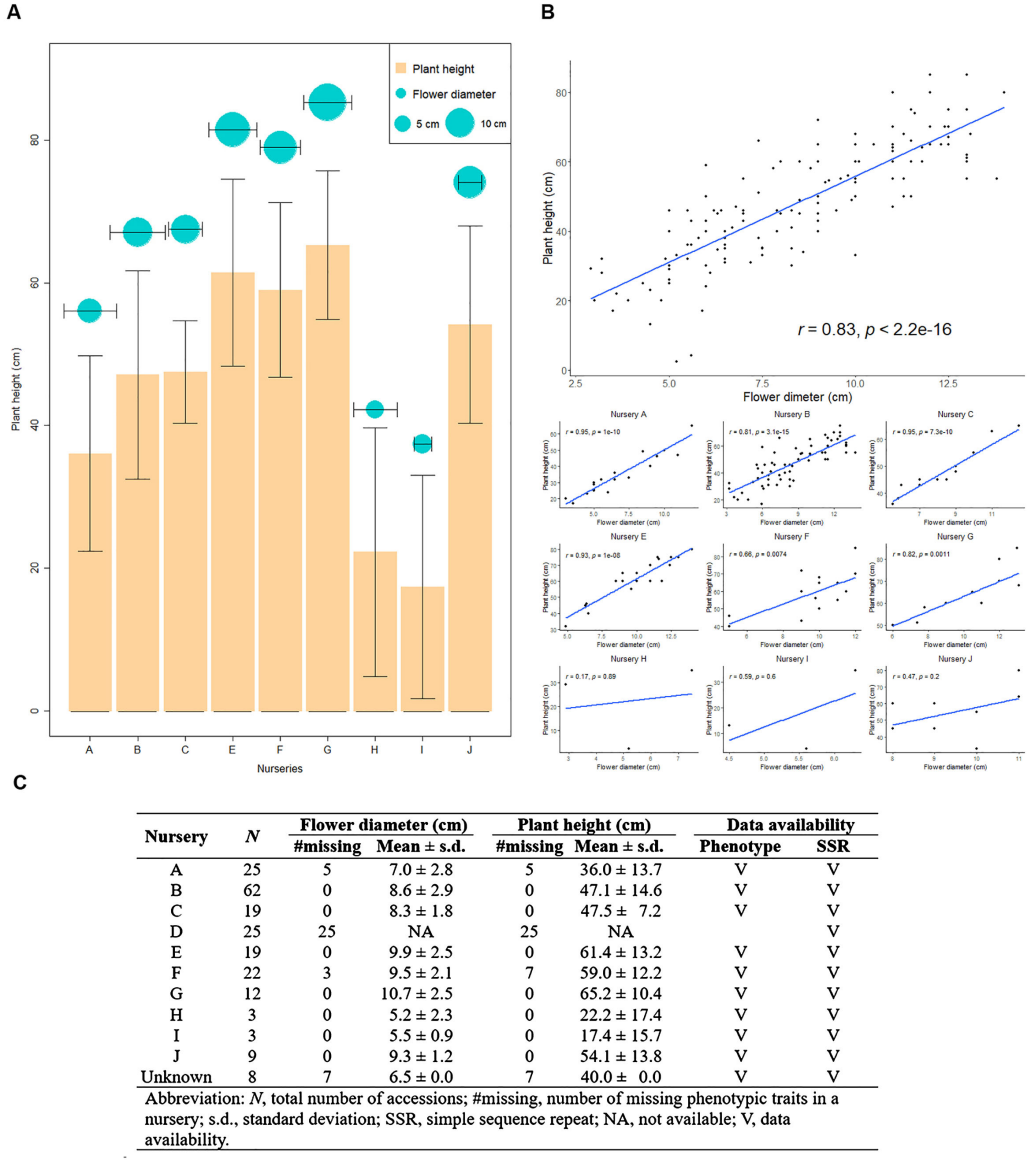
Overview of Taiwanese Phalaenopsis orchids germplasm across nurseries. **(A)** Depicts the central tendency and dispersion of phenotypic traits across various nurseries. The orange bar and cyan circle represents plant height and flower diameter, respectively. **(B)** A scatterplot illustrating the correlation between plant height and flower diameter for all nurseries, with each black dot representing an individual accession. **(C)** Provides descriptive statistics detailing plant height and flower diameter in different nurseries.

We constructed the CC from a pool of 207 orchid commercial cultivars, obtained from ten different orchid nurseries. These cultivars were characterized by two phenotypic traits, plant height and flower diameter, as well as genotypic data from eight SSR markers, including 65 SSR alleles (**Figure 4**). A significant correlation was found between 11 SSR alleles and plant height, with correlation coefficients ranging from -0.33 to 0.24 (p-value< 0.05), and between 12 SSR alleles and flower diameter, with coefficients ranging from -0.37 to 0.34 (p-value< 0.05). Overall, approximately 18% of the SSR alleles were significantly associated with these flower traits; however, the relatively low correlation coefficients suggest that the genomic regions identified by these SSRs are not closely linked to the loci influencing the studied flower traits. The modest correlations observed highlight the need to consider broader genomic regions to enhance the accuracy of genetic diversity estimation and trait association in these orchid varieties. In **Figure 4**, we illustrated the central tendency and dispersion of these two phenotypes, along with the correlation between them. Notably, nurseries G, E, F, and J showed higher values for plant height and flower diameter, while nurseries H and I exhibited lower values. Nurseries B, C, and A displayed intermediate values for both traits (**Figures 4A, C**). There was a significantly positive correlation between plant height and flower diameter (**Figure 4B**) across all nurseries (*r* = 0.83, *p*-value< 2.2×10^-16^) and within individual nurseries (*r* = 0.66~0.95, with *p*-value ranging from 7.4×10^-3^ to 3.1×10^-15^).

### Addressing missing data in phenotypic traits

The missing rate for flower diameter was 19.3%, and for plant height, it was 21.3% (**Figure 2A**). No missing data was observed in SSR alleles. The presence of missing phenotypes underestimated the genetic distance, hindering the exploration of population structure within the ES. Consequently, the collection of core accessions may be biased.

Importantly, no statistically significant difference was found between incomplete data (i.e., observed values only) and complete data (i.e., observed and imputed values) for each phenotype, as evidenced by *p*-values greater than 0.05 (**Figure 2C**; **Supplementary Table 3**). A consistently significant correlation was observed between plant height and flower diameter in both the complete data (*r* = 0.73, *p*-value< 2.2×10^-16^) and the incomplete data. This demonstrates that imputed phenotypes retain their characteristic behavior and correlation pattern.

### Population structure and genetic diversity analysis

To investigate the population structure and familial relatedness of the 207 orchid commercial cultivars, we utilized the weighted *k*-means clustering algorithm on the complete dataset. This approach classified the orchid accessions into seven distinct clusters (**Figure 2D**). We calculated genetic distances and measure dissimilarities between each pair of germplasm accessions using our proposed MGD algorithms, revealing a wide range of genetic distances among the accessions, spanning from 0.01 to 6883.6, with an average value of 508.9. The average genetic distances within clusters ranged from 3.4 to 67.8, while the average genetic distances between clusters ranged from 351.1 to 693.6. These results indicate that accessions within the same cluster exhibit close genetic distances, suggesting similarities in their phenotypic features. Conversely, accessions from different clusters exhibit greater genetic diversity, indicating differences in their phenotypic characteristics. This analysis underscores the broad range of diversity in characteristic features among orchids, including variations in flower size and height, encompassing the spectrum from the smallest to the largest blooms (**Figure 2E**).

### Selection and analysis of core collection

**Figure 5:**
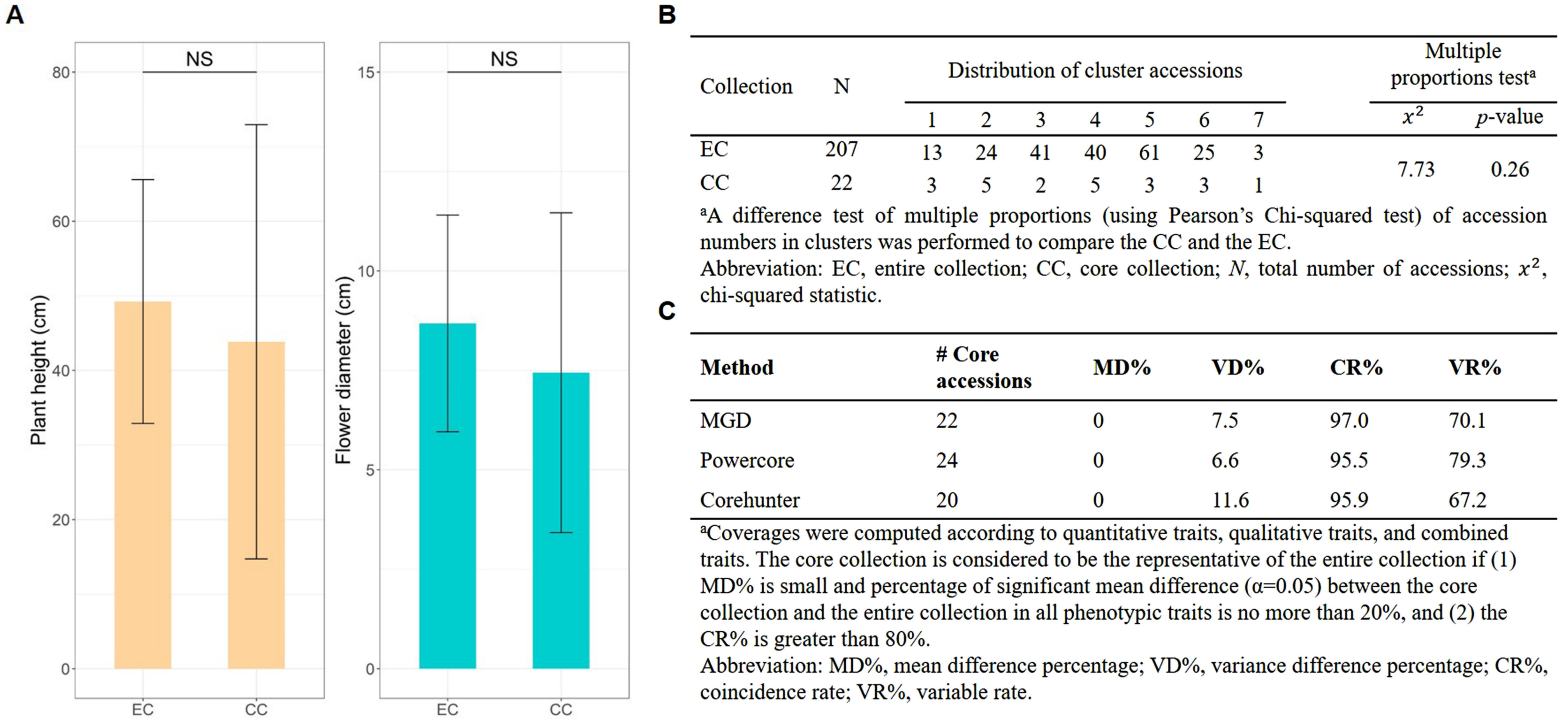
Statistical assessment of EC and CC. **(A)** Tests comparing the phenotypic traits distributions between CC and EC. **(B)** Proportions assessments of accessions across clusters between CC and EC. NS indicates non-significance. **(C)** Trait difference percentage evaluation between CC and EC.

As a result, a CC consisting of 22 orchid accessions (**Table 1**) was chosen from a pool of 207 germplasm accessions. The selection was based on their genetic distances calculated using our proposed MGD algorithms through the 2-step ‘P+G strategy’. These core accessions were evenly distributed among seven clusters (see the top of **Figure 3B**), indicating a consistent distribution of accession numbers across the clusters in the EC (**Figure 3**). Statistical analysis revealed no significant differences (*p*-value = 0.26) in multiple proportions between the CC and the EC (**Figure 5B**). This demonstrates that our MGD algorithms are capable of capturing unique characteristics and preserving genetic variability from the EC. Furthermore, there were no noticeable variations (*p*-values > 0.05) and no discernible differences (*p*-values > 0.05) between the CC and the EC regarding flower diameter and plant height, as supported by the data presented in **Figure 5A** and **Table 2**. Hence, these findings suggest that the selected core accessions adequately represent the complete range of variation in flower diameter and plant height present within the EC.

**Table 1:** A core collection comprising twenty-two accessions was selected based on the MGD.

Varieties^a^	Nurseries	Location	Cluster no.^b^	Plant height	Flower diameter
Phal.Equestris	H	Tainan	2	29.1	2.9
OX1233	E	Tainan	4	80.0	14.0
A7524	B	Changhua	3	55.0	13.8
A9333	B	Changhua	1	20.0	3.9
A7403	B	Changhua	2	25.0	4.3
A8591	B	Changhua	1	20.0	4.8
OX1701	E	Tainan	4	70.0	12.4
A10362	B	Changhua	4	65.0	12.3
OX1560	E	Tainan	2	32.0	4.9
OX1408	E	Tainan	4	74.0	11.6
K71303	G	Tainan	4	65.0	10.5
F89320	C	Pingtung	6	38.0	5.8
A9633	B	Changhua	1	17.0	5.9
A5724	B	Changhua	2	28.0	6.1
A6535	B	Changhua	5	46.0	6.2
OX1499	E	Tainan	5	46.0	6.4
A9302	B	Changhua	6	41.0	6.7
A10045	B	Changhua	5	47.0	6.8
A10731	B	Changhua	2	31.0	7.2
VM8400	G	Tainan	3	58.0	7.8
A8521	B	Changhua	6	34.0	8.6
Phal.Bellina	H	Tainan	7	2.4	5.2

^a^A core collection of 22 accessions was selected from a pool of 207 orchid commercial cultivars sourced from 10 diistinct orchid nurseries. ^b^Cluster analysis was conducted using the weighted k-means clustering algorithm.

**Table 2:** Summary statistics and difference test of phenotypic traits between the CC and the EC.

	Flower diameter		Plant height	
	EC	CC	EC	CC
Number of accessions	207	22	207	22
Summary statistics:				
Minimal value	2.9	2.9	2.4	2.4
Maximal value	14.0	14.0	85.0	80.0
Range	11.1	11.1	82.6	77.6
Mean	8.7	7.6	49.2	42.0
Standard deviation	2.7	3.3	16.3	20.6
Coefficient of variation (%)	31.4	43.4	33.2	49.1
Difference test:				
Homogeneity test for variance	*p*-value = 0.44		*p*-value = 0.08	
Difference test for mean	*p*-value = 0.10		*p*-value = 0.06	

EC, entire collection; CC, core collection.

a Core collection was constructed by using the modified genetic distance (MGD) method.

^b^The Levene’s test was first used to test homogeneity of variance, followed by Student’s t-test for mean difference among two collections.

### Evaluation of core accessions effectiveness

**Figure 6:**
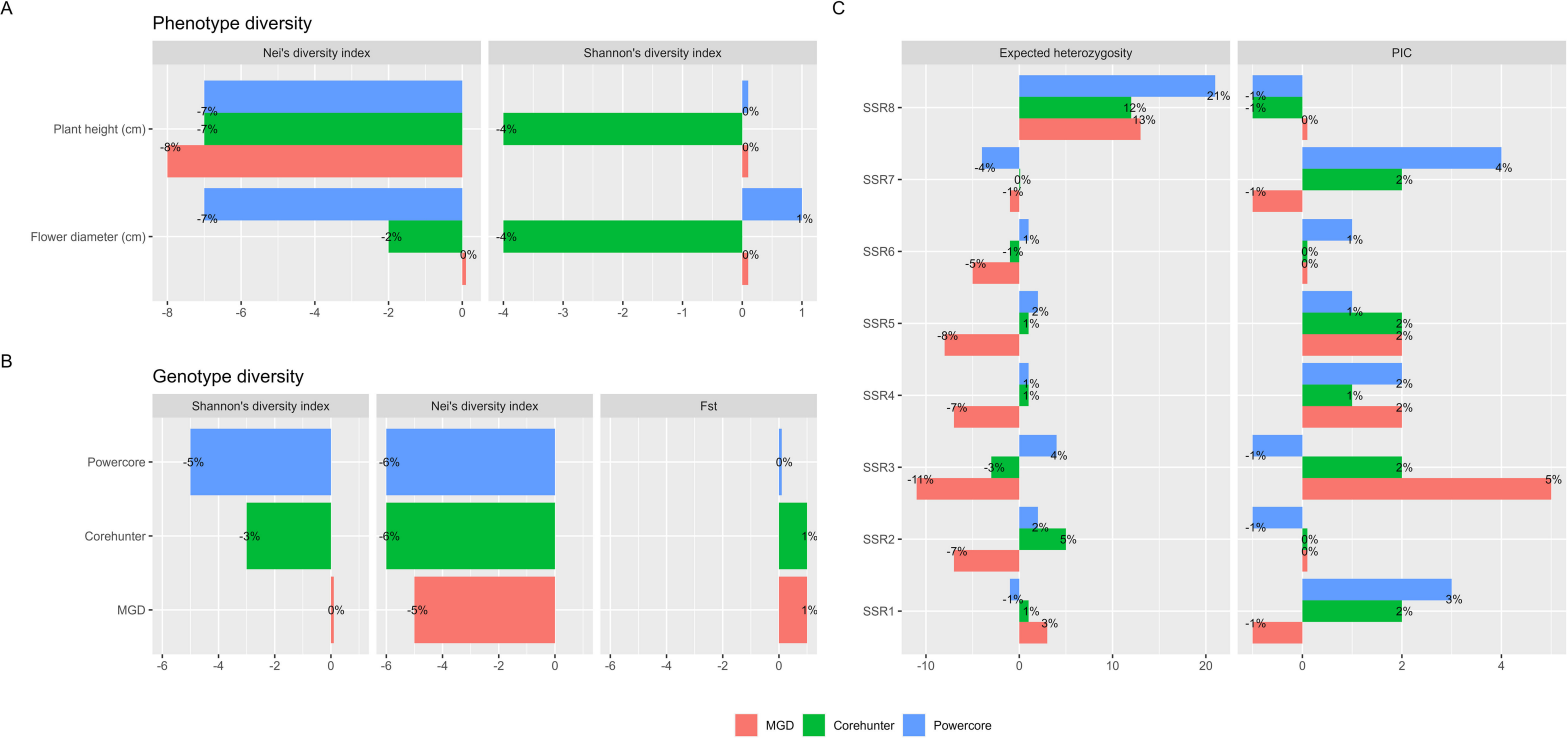
Diversity comparison between CC and EC. **(A)** Phenotypic diversity change measured by Shannon’s index and Nei’s diversity index. **(B)** Genotypic diversity change measured by Shannon’s index, Nei’s diversity index and fixation index (F_ST_). **(C)** Genetic diversity change measured by polymorphic information content (PIC) and expected heterozygosity.

We evaluated the effectiveness of the selected core accessions by analyzing changes in diversity, central tendency, and variability of both phenotypic and genotypic data between the CC and the EC. These evaluations are summarized in **Table 3** and **Figure 6**.

Table 3Changes in diversity between the CC and the EC.

**Table:** 

(A) Changes in phenotypic diversity								
Diversity	EC		CC^a^					
			MGD		Powercore		Corehunter	
	H′	Nei’s	H′	Nei’s	H′	Nei’s	H′	Nei’s
Overall diversity:								
Phenotypic diversity	0.98	0.89	0.98	0.84	0.93	0.83	0.95	0.83
Diversity changes			0	-0.05	-0.05	-0.06	-0.03	-0.06
Preservation			^b^V					
Loss in diversity richness				V		V		V
Loss in diversity evenness					V		V	
Diversity of individual traits:								
Flower diameter (cm)	0.98	0.73	0.98	0.73	0.99	0.66	0.94	0.71
Diversity changes			0	0	+0.01	-0.07	-0.04	-0.02
Preservation			V	V	V			
Loss in diversity richness						V		V
Loss in diversity evenness							V	
Plant height (cm)	0.90	0.81	0.90	0.73	0.90	0.74	0.86	0.74
Diversity changes			0	-0.08	0	-0.07	-0.04	-0.07
Preservation			V	V	V			
Loss in diversity richness						V		V
Loss in diversity evenness							V	

EC, entire collection; CC, core collection; H′, Shannon-Weaver diversity index; Nei’s, Nei’s diversity index; MGD, modified genetic distance.

a A total of 22, 24, and 20 core accessions were selected by using MGD, Powercore, and Corehunter.

b V represented Yes in this table.

**Table:** 

(B) Changes in genetic diversity								
Method	EC		Diversity changes in CC^a^					
			MGD		Powercore		Corehunter	
	PIC	H_exp_	PIC	H_exp_	PIC	H_exp_	PIC	H_exp_
Overall diversity:								
Genetic diversity	0.22	0.83	+0.01	+0.03	+0.01	-0.01	+0.01	+0.01
Preservation			V	V	V		V	V
Loss in diversity identification								
Loss in genetic diversity						V		
Diversity of individual SSR:								
SSR1	0.14	0.83	-0.01	+0.03	+0.03	-0.01	+0.02	+0.01
SSR2	0.32	0.65	0	-0.07	-0.01	+0.02	0	+0.05
SSR3	0.28	0.80	+0.05	-0.11	-0.01	+0.04	+0.02	-0.03
SSR4	0.16	0.81	+0.02	-0.07	+0.02	+0.01	+0.02	+0.01
SSR5	0.26	0.73	+0.02	-0.08	+0.01	+0.02	+0.02	+0.01
SSR6	0.18	0.81	0	-0.05	+0.01	+0.01	0	-0.01
SSR7	0.17	0.82	-0.01	-0.01	+0.04	-0.04	+0.02	0
SSR8	0.32	0.07	0	0.13	-0.01	+0.21	-0.01	+0.12

EC, entire collection; CC, core collection; PIC, polymorphic information content; H_exp_, expected heterozygousity; SSR, simple sequence repeat; MGD, modified genetic distance.

a A total of 22, 24, and 20 core accessions were selected by using MGD, Powercore, and Corehunter.

b V represented Yes in this table.

The 22 core accessions, carefully selected through the 2-step P+G framework and MGD algorithms, exhibited noteworthy phenotypic diversity across various traits, with an overall diversity index (H′) of 98% and Nei’s diversity index reaching 84%. Specifically, traits like flower diameter and plant height, exhibited substantial diversification within these core accessions, recording H′ values of 98% and 90% respectively, and Nei’s diversity indices of 73%. When contrasted with the EC as delineated in **Table 3A**, the core accessions effectively maintained diversity evenness, with H′ showing no significant changes while experiencing a minor reduction in Nei’s diversity richness (**Figure 6A**).

Regarding genetic diversity, measured through PIC values, expected heterozygosity, fixation index (F_ST_), H′ and Nei’s diversity index, the CC exhibited a slightly higher PIC value of 0.23 (ranging from 0.13 to 0.33 across individual SSR markers) compared to the EC, which had a PIC value of 0.22 (ranging from 0.14 to 0.32 in individual SSR markers) (see **Table 3B**). This difference indicates a subtle yet notable enrichment of allelic diversity within the CC at these genetic markers (**Figure 6C**), suggesting a distinct genetic richness compared to the EC. In terms of expected heterozygosity, the CC constructed by the MGD algorithm showed a slight decrease, ranging from 0.86 to 0.20 (**Figure 6C**). Additionally, the MGD algorithm achieved an H’ of 0.93, a Nei’s diversity index of 0.79, and an F_ST_ value of 0.02. Apart from the slight loss in Nei’s diversity index, the other indicators suggest that the CC constructed by the MGD algorithm can preserve the diversity of the EC (**Figure 6B**), and it increases genetic differentiation or variability among individuals within the core accessions compared to the EC at these loci.

Noteworthy is the superior performance of the 22 core accessions chosen via the 2-step P+G framework and MGD algorithms over selections made by other methodologies like Powercore (24 core accessions) and Corehunter (20 core accessions). Our CC not only retained diversity evenness in phenotypic traits but also demonstrated a minimal decrease in diversity richness, outperforming other methods (please refer to **Table 3A**). Similarly, in genetic diversity assessment, our CC showcased enhanced diversity identification and preserved genetic variation, surpassing alternative methodologies (**Table 3B**). These findings highlight the distinctive genetic richness within the CC compared to the EC, providing valuable insights into the genetic diversity and potential selection of genetically diverse individuals for breeding or conservation purposes.

In addition, we found no significant changes in the central tendency of phenotypes (MD% = 0%) and only slight changes in phenotypic variability (VD% = 7.5%) between the CC and the EC. Moreover, there was a highly consistent phenotypic variation (CR% = 97% and VR% = 70.1%) was observed between the CC and the EC.

### Validation of core collection based on pedigree background

Confirming that the CC contains the most diversity from the EC is crucial. We employed various methods to validate the fitness of our CC. Another aspect of this validation involves assessing the appropriateness of the CC based on pedigree background. To achieve this, we traced back the ancestors of 79 cultivars from the EC and estimated the genetic background of 17 native *Phalaenopsis* species among them as a percentage (**Figure 6A**). Among these, twelve cultivars were included in the CC for this study (**Figure 6B**). The ancestor *P. amabilis* exhibited the highest contribution to the genetic background, at 30%, followed by *P. rimestadiana* (20%) and *P. aphrodite* (11%), collectively accounting for approximately 60% of the diversity based on unique or distinct genetic backgrounds. Moreover, the genetic background was largely explained in most of the cultivars, with cumulative percentages ranging from 88% to 100%, except for one variety (A8591) with 50% (**Supplementary Table 4**). Furthermore, we identified a strong correlation, with an R^2^ value of 0.91 (*p*-value = 5.9×10^-7^), between the known pedigrees of the 79 cultivars from the EC and the known pedigrees of the 12 cultivars within the CC (**Figure 6C**). Taken as a whole, the 22 core accessions identified through the proposed 2-step ‘P+G strategy’ and MGD algorithms effectively preserve genetic diversity from the EC and could serve as excellent representatives of the EC.

## Discussion

The present investigation marks the inaugural documentation of a CC specifically for Phalaenopsis orchids in Taiwan (**Supplementary Table 1**). Developing a reliable core collection is essential for safeguarding genetic diversity and capturing indispensable traits of a species. Our study used an innovative methodology combining phenotypic and genotypic data to curate a core collection of 22 orchid accessions (**Table 1**). The application of the two-step ‘P+G strategy’ and the MGD algorithm confirmed the value and reliability of these core accessions. This endeavor highlights key aspects of establishing and evaluating a core collection, which holds promise for breeding commercially desirable varieties and advancing further research in the field.

Handling missing phenotypic data was crucial for ensuring the accuracy of our core collection. Discussions concerning the limitations and challenges arising from missing data have been detailed elsewhere (Bateman and Rudall, 2023). In this study, we used multiple imputation techniques within a Bayesian framework to address missing values, maintaining the integrity of the phenotypic data (**Figure 2**; **Supplementary Table 3**). Previous studies have shown the efficacy of the MI algorithm, particularly with small sample sizes or high rates of missing data (Cheema, 2014). Statistical analyses revealed no significant differences between observed and imputed values, validating our imputation approach.

Orchids are known for their diverse and stunning flowers, which come in various shapes, colors, sizes. Flower diameter and plant height provide valuable information about the growth and characteristics of orchid plants, contributes to the overall beauty and appeal of orchids. Flower diameter is an important trait used to assess the size, symmetry, and overall appearance of orchid flowers. Plant height can vary widely among orchid species, with some orchids being small and compact (showed as cluster 7 in **Figures 2D, E**) while others are tall and erect (showed as cluster 4 in **Figures 2D, E**). Flower diameter and plant height can vary significantly among different orchid species and even within the same species, and thus they are important parameters used to assess the visual appeal, size, overall appearance, and growth patterns of orchid flowers. The genetic diversity observed among the clusters implies that the orchids exhibit a wide spectrum of characteristic features, including flower size and plant height. Orchid enthusiasts and collectors often appreciate and value orchids with large, well-formed flowers. Overall, flower diameter is a significant parameter in assessing and appreciating the visual impact of orchid flowers, and it plays a role in various aspects of orchid cultivation, research, and hybridization.

In this investigation, the examination of the population structure elucidated substantial genetic diversity among individual orchid accessions, elucidating inherent variations in their genetic makeup. Employing clustering analysis, our orchid accessions underwent categorization into seven distinct clusters, visually illustrating the diversity and mutual relationships among these clusters based on their genetic profiles (refer to **Figure 5**). These clusters demarcate unique lineages or populations within our orchid collection, where accessions within a cluster demonstrate heightened genetic similarity among themselves compared to accessions in different clusters. Remarkably, a notable discrepancy in genetic distances was observed between accessions from distinct clusters (inter-cluster accessions) as opposed to those within the same cluster (intra-cluster accessions) (**Supplementary Figure 1**). This discrepancy suggests that each cluster potentially harbors distinct traits or variations in pivotal orchid characteristics, thereby significantly contributing to the overall observed diversity within our collection. Specifically, our analysis delved into the distribution patterns of plant height and flower diameter within each cluster, revealing seven distinct characteristics. This scrutiny underscored both the proximity among accessions within clusters and the pronounced differences between accessions across separate clusters, emphasizing the diversity spectrum of phenotypic features, particularly in flower size and plant height. Collectively, these findings corroborate the efficacy and reliability of our clustering methodology.

Evaluation of the core accessions showed they maintained substantial phenotypic diversity, with minor reductions in trait diversity compared to the EC. Traits like flower diameter and plant height exhibited significant diversification, with high H’ values and Nei’ s diversity indices (**Table 3A**). Genetically, the core collection displayed a higher PIC value compared to the EC, indicating enriched allelic diversity within specific markers (**Table 3B**). Statistical comparisons confirmed the core accessions’ adequacy in representing the full range of variation in flower diameter and plant height (**Table 2**; **Figure 6A**). The selected core accessions outperformed other methods by preserving diversity evenness and enhancing genetic diversity identification.

The applied selection strategy, specifically the 2-step ‘P+G strategy’ incorporating MGD algorithms, effectively captured distinct and uncommon traits, particularly those of rare characteristics, while simultaneously preserving genetic variability within the CC to ensure a comprehensive representation of the EC (refer to **Figure 2**). This approach adeptly allocated 22 core accessions across seven clusters (**Table 1**), revealing a statistically nonsignificant difference between the CC and the EC (**Figure 5B**). This outcome ensures an equitable representation of diverse genetic backgrounds within the selected subset, surpassing the representation achieved by individual ‘P-only’ or ‘G-only’ strategies. The observed results underline the capability of the 2-step ‘P+G strategy’ in encapsulating diverse and rare traits within the CC. In contrast, the ‘G-only strategy’ in selecting core accessions fails to encompass the entirety of the rare characteristics, resulting in the exclusion of the 7^th^ cluster from the CC, likely due to constraints inherent in the SSR markers utilized (e.g., SSR markers are more susceptible to the effects of species formation and evolutionary processes). Despite the informative nature of SSR markers, their limitations may arise from a potential lack of resolution or specificity to sufficiently capture the distinct genetic variations defining rare characteristics in the population. These markers may predominantly emphasize prevalent or common genetic features, potentially neglecting the intricacies and subtleties inherent in rare traits, consequently leading to the inability to discern the 7^th^ cluster. To elaborate, although genomic information was identified across 65 loci utilizing 8 SSR markers among these orchid materials, the regions harboring SSR diversity might not have been distinguished between the 7^th^ cluster and the remaining clusters in this investigation. Furthermore, the ‘P-only strategy’ was proficient in detecting rare features while overlooking other significant traits, possibly due to its reliance solely on phenotypic data. The analogous situation was observed in the traditional P+G strategy. Phenotypic traits, influenced by genetic and environmental factors, may exhibit conspicuous rare characteristics without always directly correlating with underlying genetic variations. Consequently, the ‘P-only strategy’ might identify certain evident rare characteristics but could potentially overlook or fail to correlate with other crucial genetic variations that do not prominently manifest in observable phenotypes. Addressing the complexities involved in identifying rare characteristics and ensuring comprehensive diversity representation within a population requires integrating diverse data types encompassing both phenotype and genotype, utilizing advanced algorithms like the 2-step ‘P+G strategy’ and MGD. This integration enables a more accurate capture of the entire genetic variability spectrum, particularly the elusive rare traits, during the core accessions selection process.

Evaluation of the effectiveness of the core accessions revealed that they phenotypically maintained substantial diversity and evenness, showcasing only minor reductions in overall trait diversity and individual trait diversity richness when compared to the EC. Specifically, traits such as flower diameter and plant height exhibited significant diversification, reflecting H′ values of 98% and 90% respectively, and Nei’s diversity indices of 73% (refer to **Table 3A**). Genetically, the CC displayed a slightly higher PIC value compared to the EC (**Table 3B**), suggesting a nuanced enrichment of allelic diversity within specific genetic markers. Nei’s gene diversity also demonstrated a marginally higher value in the CC, indicating increased genetic differentiation or variability compared to the EC at these loci (**Table 3**; **Figure 6**). Moreover, statistical comparisons between the CC and the EC (**Table 2**; **Figure 6A**) confirmed the adequacy of the chosen core accessions in encompassing the complete range of variation in flower diameter and plant height present within the larger germplasm accessions. Remarkably, the 22 selected core accessions outperformed other selection methods such as Powercore and Corehunter by preserving diversity evenness in phenotypic traits and showcasing minimal reduction in diversity richness, along with enhanced genetic diversity identification compared to alternative methodologies. These findings feature the distinct genetic richness within the CC, offering valuable insights into genetic diversity preservation for breeding or conservation purposes.

Validating the fitness of the diversity representation within the CC based on pedigree background offered an additional layer of confirmation to comprehend the composition of genetic diversity from different ancestors. Among the 12 selected cultivars traced from the 79 cultivars in the EC, significant genetic contributions were evident from several key ancestors like *P. amabilis*, *P. rimestadiana*, and *P*. *Aphrodite*. These ancestors collectively contributed significantly to the genetic diversity, accounting for approximately 61% of the unique genetic backgrounds, indicating their prominent role in the breeding process. These three species share white flower color but differ in flower diameter, and white flowers are a critical trait in Taiwanese breeding practices. This explains their significant contribution among the 17 distinct ancestors. Tracing the genetic backgrounds of these cultivars and establishing strong correlations between known pedigrees further substantiated the representativeness of the core accessions in preserving the genetic diversity inherent in the EC. Notably, most cultivars in the CC exhibited well-explained genetic backgrounds, emphasizing the comprehensive representation achieved.

**Figure 7:**
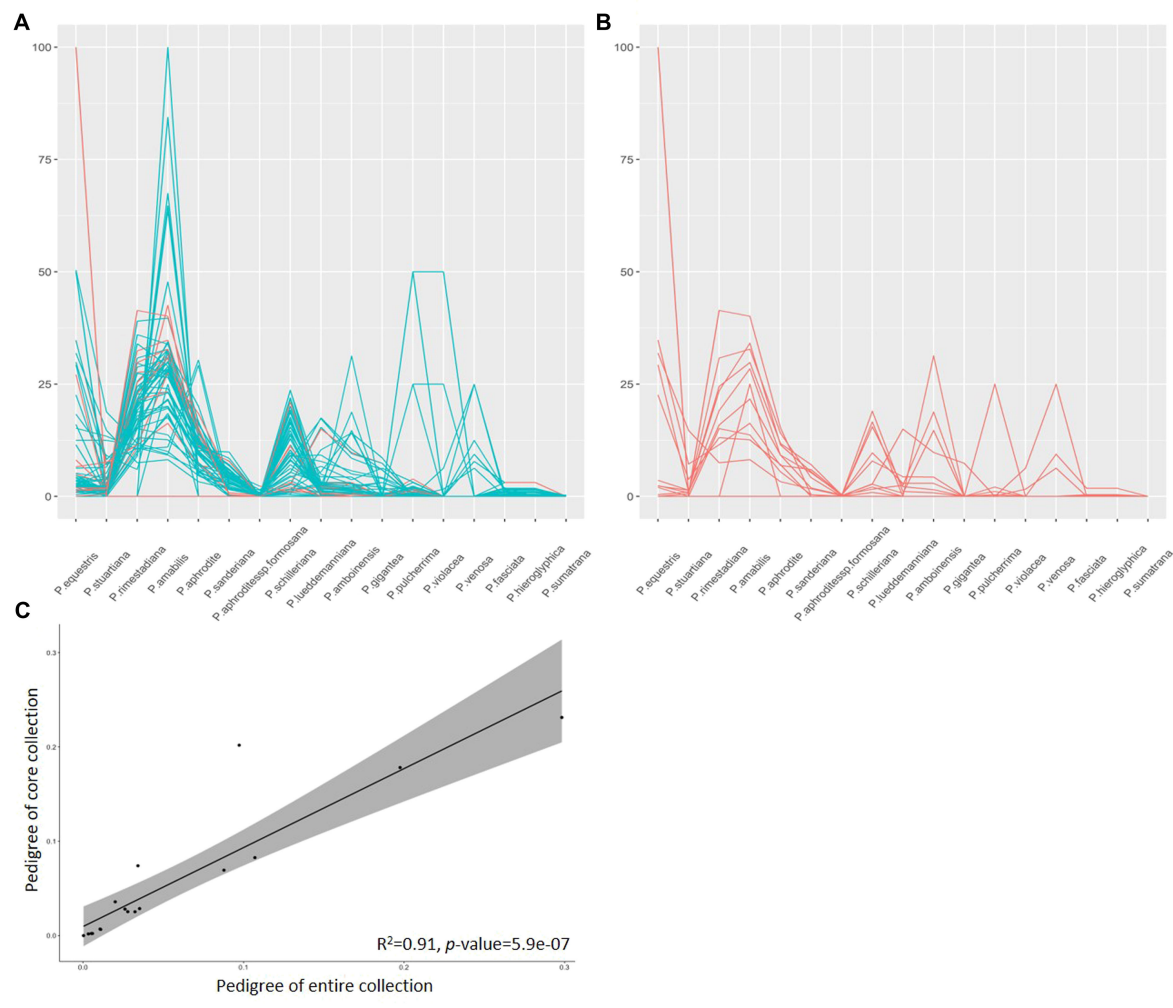
Pedigree background analysis of orchid cultivars. **(A)** Genetic background identification percentage for ancestors of 79 cultivars from the EC. **(B)** Genetic background identification percentage for ancestors of 79 cultivars from the CC. **(C)** Correlation of pedigrees between the EC and CC. Cyan and red lines represent the percentage of identified ancestors for the 79 cultivars, respectively.

The statistical perspective has confirmed the CC’s capacity to effectively represent the original germplasms. However, an additional validation through genealogical assessment, examining genetic background similarity based on pedigrees, is crucial for providing concrete data illustrating the extent of similarity between the CC and the EC. Identification of 17 distinct orchid genetic backgrounds among the 79 cultivars within the original germplasms was conducted, with authentication performed using OrchidWiz (https://www.orchidwiz.com/). Among the 12 CC cultivars mapped back through genealogy (refer to **Figure 7**), 16 genetic backgrounds were identified, excluding *P*. *sumatrana* (see **Supplementary Table 4**). The correlation between the CC and the EC based on the genealogy ratio further confirmed a strong alignment (*r* = 0.976) with statistically significant results (*p*-value = 2.16×10^-11^), reinforcing the credibility of our selection process. Additionally, a strong goodness of fit (*R* ^2 = ^0.91) was observed, indicating a high level of genetic diversity based on pedigree similarity between the CC and the EC. This result enhances our confidence in the methodology employed in this study. Furthermore, variety A8591, explaining 50% of its genetic background based on the pedigrees of 17 distinct ancestors, implies the presence of additional genetic diversity beyond the scope of these 17 genetic backgrounds. The inclusion of this variety in our CC supports the effectiveness of our method in capturing a broad spectrum of diversity within core accessions. These robust statistical outcomes confirm again the CC’s adept preservation of diverse genetic materials, establishing it as an excellent representative subset of the entire germplasm collection.

This study is subject to certain limitations. Firstly, previous studies (Kumar et al., 2016; Mahmoodi et al., 2021) advocate for the integration of phenotypic and genotypic data to construct a CC that comprehensively encompasses maximum variability within germplasms. The utilization of SSR markers may not depict a complete genetic diversity within the EC due to a limited number of markers, low resolution, bias towards highly polymorphic regions, and the impacts of species formation and evolutionary processes (Lee et al., 2016). To address this limitation associated with the use of SSR markers and to provide a more comprehensive understanding of distinct and rare features within the CC, we implemented a two-step ‘P+G strategy’ incorporating MGD algorithms. The rationale behind recognizing the limitation of SSR marker is to justify the adoption of this strategy. Secondly, the Nei’s gene diversity of SSR is relatively lower, indicating reduced genetic diversity among diverse samples. This decline may be attributed to the exclusion of regions with genetic variance by the employed SSR markers. Additionally, our analysis of pedigree backgrounds reveals that approximately 60% of the CC’s lineage can be traced back to three ancestral sources, potentially contributing to the observed decline in genetic diversity associated with SSR. Market-oriented breeding practices have the potential to influence breeder choices, directing attention towards popular external phenotypic traits, such as large white flowers, in demand within the Taiwanese market. This shift in focus has implications for genetic diversity, potentially leading to a decline. In contrast, single nucleotide polymorphism (SNP) markers are deemed more advantageous for the analysis of genetic variation, owing to their higher overall count and widespread distribution across the genome (Li et al., 2010), especially in the context of agronomic and morphological traits (Lee et al., 2016). To overcome this limitation, our current efforts are directed towards an ongoing whole-genome genotyping project, encompassing a coverage of approximately 16X for the utilized materials. This undertaking is anticipated to produce a plethora of genome-wide SNPs, potentially alleviating the limitations associated with the deployed SSR markers.

In conclusion, the 22 core accessions, meticulously selected through the 2-step ‘P+G strategy’ and MGD algorithms, effectively retain genetic diversity and critical phenotypic traits, positioning them as comprehensive representatives of the broader orchid germplasm collection. This CC stands as a valuable resource for future studies in orchid breeding programs, conservation efforts, and genetic improvement for sustainable agricultural practices. Exploration using a broader spectrum of markers or additional genetic diversity metrics would provide a more comprehensive portrayal of the genetic composition within these collections.

## Data Availability

The datasets presented in this study can be found in online repositories. The names of the repository/repositories and accession number(s) can be found below: https://doi.org/10.5061/dryad.fn2z34v2z.
