# Supplementary material for: Genetic profiles and phenotypic patterns in Taiwanese Phalaenopsis orchids: a two-step phenotype and genotype strategy using modified genetic distance algorithms
Source: Front Plant Sci. 2024 Sep 11;15:1416886. doi: 10.3389/fpls.2024.1416886 (PMC11422071; doi:10.3389/fpls.2024.1416886)
Supplement: Supplementary file 1 [file DataSheet1.docx]

Supplementary Material

# Supplementary Figures and Tables

**
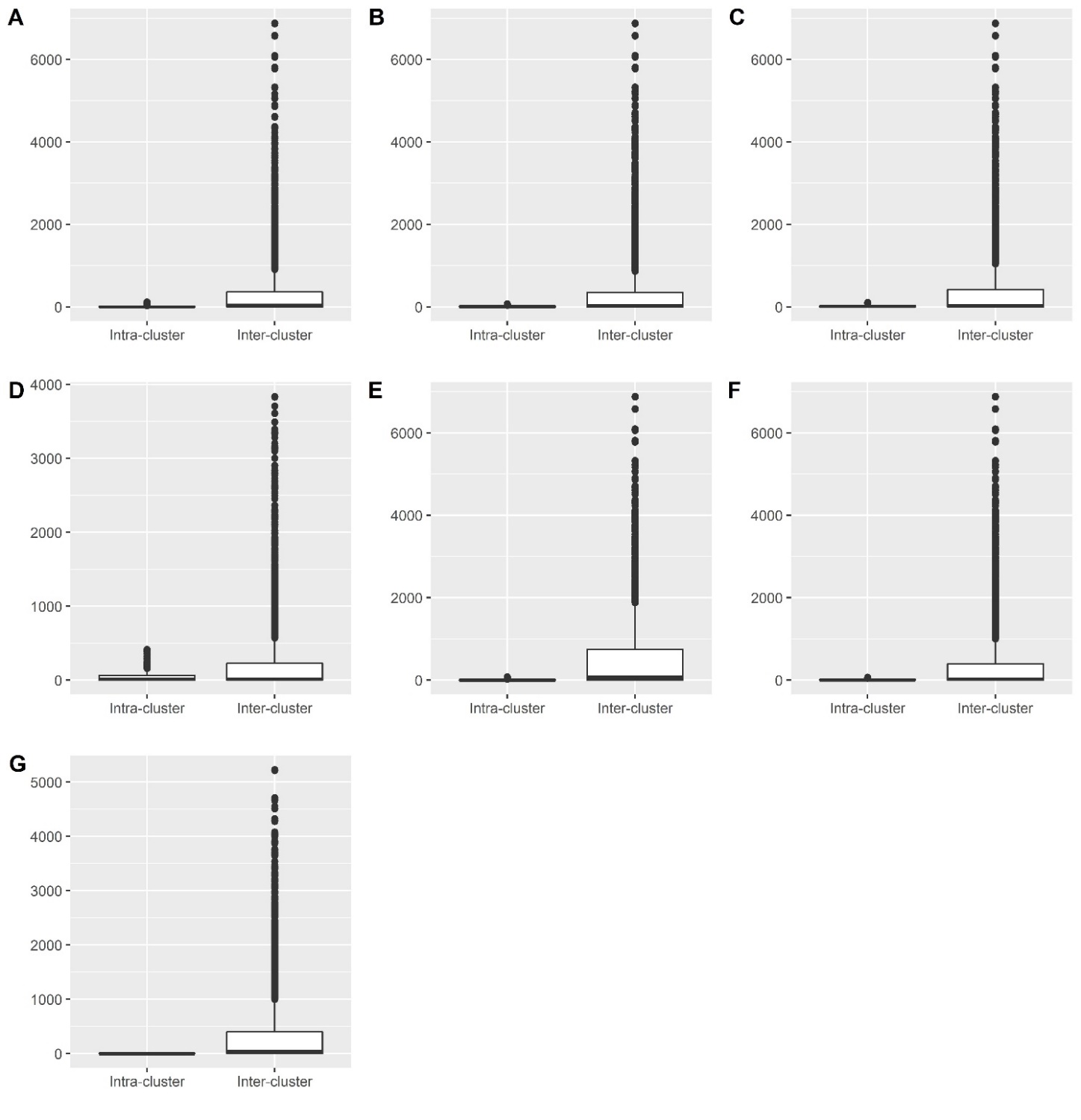
**

**Supplementary Figure 1.** Genetic distance among accessions from distinct clusters (inter-cluster accessions) and within clusters (intra-cluster accessions) for cluster 1 through 7 (A-G). The genetic distance were calculated using the modified genetic distance (MGD) algorithm, which employs the Euclidean distance for quantitative traits and the inverse occurrence frequency (IOF) measure for qualitative data.

**Supplementary Table 1.** Summary information of orchid cultivars collected from distinct orchid nurseries in Taiwan.

| **Orchid nursery** | **Location** | **Number of orchid cultivars** |
| --- | --- | --- |
| A | Pingtung | 25 |
| B | Pingtung | 62 |
| C | Tainan | 19 |
| D | Tainan | 25 |
| E | Pingtung | 19 |
| F | Taichung | 22 |
| G | Changhua | 12 |
| H | Tainan | 3 |
| I | Tainan | 3 |
| J | Taichung | 9 |
| Unknown |  | 4 |
| Total |  | 207 |

**Supplementary Table 2.** The SSR primer sequences applied in this study.

| SSR marker | Forward primer (5’-3’) | Reverse primer (5’-3’) | Tm (℃) |
| --- | --- | --- | --- |
| SSR1 | TCTTTGTGTGTGTGTGCGTG | GTGTCCCGTAGACTTCCCG | 55 |
| SSR2 | AACGCATTTGGCTCAGGC | AGATTGTTCGGACTACAAGGCG | 55 |
| SSR3 | GGGATGGAGGGATTTGAGAT | AAGCACAGGAAACGCAAACT | 55 |
| SSR4 | GATTACTATTTTGATGTGCTCTGC | CGTCACCGATTACAGCACAT | 55 |
| SSR5 | CCGCTCTTCCAGTTTCGTT | AAATCATCTTAGGAGCACCATCA | 55 |
| SSR6 | CTCATTCGGTCAGGCATTTC | AGCTGCTTCCCAAGTGATTC | 55 |
| SSR 7 | TGGTCTCTGTCGTCACTTGG | AAGAATTACACCGCCGATCA | 55 |
| SSR 8 | GAGCCCGATCACAACGACC | GTGACTCCGTTCCATGCCTC | 55 |

**Supplementary Table 3.** A difference test of phenotypic traits between the complete (observed and imputed values) and the incomplete (observed values) phenotypes.

| **Phenotypic trait** | **Incomplete phenotypes^a^**  **(Observed values)** | |  | **Complete phenotypes^b^**  **(Observed + imputed values)** | |  | **Difference test**  **(p-value)^c^** |
| --- | --- | --- | --- | --- | --- | --- | --- |
|  | **N** | **Mean±sd** |  | **N** | **Mean±sd** |  |  |
| Flower diameter (cm) | 167 | 8.7 ± 2.7 |  | 207 | 8.6 ± 2.7 |  | 0.76 |
| Plant height (cm) | 163 | 48.2 ± 16.3 |  | 207 | 48.1 ± 16.2 |  | 0.52 |
| Abbreviation: N, number of germplasms; sd, standard deviation.  ^a^Incomplete phenotypes include both observed phenotypes and some missing phenotypes. ^b^Complete phenotypes include both observed and imputed phenotypes. Multiple imputation was employed to estimate the missing phenotypes. ^c^A paired *t*-test was conducted to test the difference between complete and incomplete phenotypes. | | | | | | | |

**Supplementary Table 4.** Percentage of unique or distinct ancestors in the pedigrees (%).

| Variety | *P*. equestris | *P*. stuartiana | *P*. rimestadiana | *P*. amabilis | *P*. aphrodite | *P*. sanderiana | *P*. aphrodite ssp. formosana | *P*. schilleriana | *P*. lueddemanniana | *P*. amboinensis | *P*. gigantea | *P*. pulcherrima | *P*. violacea | *P*. venosa | *P*. fasciata | *P*. hieroglyphica | *P*. sumatrana | Total percentage |
| --- | --- | --- | --- | --- | --- | --- | --- | --- | --- | --- | --- | --- | --- | --- | --- | --- | --- | --- |
| N1414 | 50.4 | 18.8 | 11.8 | 10 | 4.6 | 2.7 | 1.4 | 0.4 | 0 | 0 | 0 | 0 | 0 | 0 | 0 | 0 | 0 | 100.1 |
| OX1249 | 2.3 | 0.8 | 19.8 | 28 | 9.7 | 1.6 | 0.3 | 1.7 | 5.5 | 14.1 | 1.6 | 2 | 0 | 9.4 | 1.6 | 1.6 | 0 | 100 |
| TWM_2 | 34.7 | 8.2 | 15.3 | 17.5 | 7 | 4.7 | 0.6 | 8.2 | 1.8 | 1.4 | 0 | 0.2 | 0 | 0 | 0 | 0 | 0 | 99.6 |
| B1470 | 0.9 | 0.5 | 22.8 | 23.2 | 10.1 | 4.2 | 0 | 9.1 | 0.9 | 0.8 | 0 | 0.8 | 0 | 25 | 0.8 | 0.8 | 0 | 99.9 |
| M3934 | 2.3 | 0.8 | 19.8 | 28 | 9.7 | 1.6 | 0.3 | 1.7 | 5.5 | 14.1 | 1.6 | 2 | 0 | 9.4 | 1.6 | 1.6 | 0 | 100 |
| A7835 | 1.5 | 1.6 | 17.3 | 19.6 | 9.9 | 3.1 | 0.1 | 8.3 | 17.3 | 9.5 | 7.4 | 0.2 | 0 | 0 | 1.9 | 0 | 0.3 | 98 |
| A11333 | 1.6 | 0.5 | 12 | 67.5 | 5.5 | 2.4 | 0.2 | 8.1 | 1 | 0.7 | 0 | 0.3 | 0 | 0 | 0 | 0 | 0 | 99.8 |
| A6371 | 12.5 | 12.5 | 11.4 | 9.4 | 29.2 | 0 | 0 | 0 | 0 | 0 | 0 | 0 | 0 | 25 | 0 | 0 | 0 | 100 |
| T2211 | 29.3 | 1 | 15.9 | 21.7 | 9.2 | 5.3 | 0.4 | 7.9 | 4.4 | 4.3 | 0 | 0 | 0 | 0 | 0 | 0 | 0 | 99.4 |
| Lee_1150 | 6.6 | 7.6 | 11.5 | 27.5 | 18.5 | 4.4 | 0 | 23.7 | 0.1 | 0 | 0 | 0 | 0 | 0 | 0 | 0 | 0 | 99.9 |
| A11595 | 1.1 | 1.1 | 11.7 | 64.7 | 5.8 | 4.5 | 0 | 10.4 | 0.3 | 0 | 0 | 0.4 | 0 | 0 | 0 | 0 | 0 | 100 |
| M1380 | 0 | 0 | 9 | 84.4 | 5 | 0 | 0 | 1.6 | 0 | 0 | 0 | 0 | 0 | 0 | 0 | 0 | 0 | 100 |
| Lee_1243 | 5.2 | 4.1 | 16.1 | 47.8 | 13.1 | 1.5 | 0.2 | 10.3 | 0.6 | 0.4 | 0.4 | 0.1 | 0 | 0 | 0.1 | 0.1 | 0 | 100 |
| A5638 | 29.3 | 1 | 15.9 | 21.7 | 9.2 | 5.3 | 0.4 | 7.9 | 4.4 | 4.3 | 0 | 0 | 0 | 0 | 0 | 0 | 0 | 99.4 |
| F89251 | 1.4 | 0.3 | 16.3 | 21.4 | 7.5 | 1.9 | 0.1 | 0.5 | 10.4 | 14.1 | 8.6 | 0 | 0 | 7.8 | 3.1 | 3.1 | 0 | 96.5 |
| Lee_1287 | 5.2 | 4.1 | 16.1 | 47.8 | 13.1 | 1.5 | 0.2 | 10.3 | 0.6 | 0.4 | 0.4 | 0 | 0 | 0 | 0.1 | 0.1 | 0.1 | 100 |
| F89154 | 1.4 | 0.3 | 16.3 | 21.4 | 7.5 | 1.9 | 0.1 | 0.5 | 10.4 | 14.1 | 8.6 | 0 | 0 | 7.8 | 3.1 | 3.1 | 0 | 96.5 |
| Lee_1288 | 5.2 | 4.1 | 16.1 | 47.8 | 13.1 | 1.5 | 0.2 | 10.3 | 0.6 | 0.4 | 0.4 | 0 | 0 | 0 | 0.1 | 0.1 | 0.1 | 100 |
| OX1671 | 1.5 | 1.6 | 22.9 | 30.9 | 11.3 | 7.8 | 0.1 | 21 | 0.7 | 0.4 | 0 | 0.7 | 0 | 0 | 0.4 | 0.4 | 0 | 99.7 |
| A7431 | 2.3 | 0.8 | 19.8 | 28 | 9.7 | 1.6 | 0.3 | 1.7 | 5.5 | 14.1 | 1.6 | 2 | 0 | 9.4 | 1.6 | 1.6 | 0 | 100 |
| OX1740 | 2.9 | 2.1 | 21.5 | 28.9 | 11.8 | 5 | 0.4 | 15.4 | 4.9 | 2.8 | 2 | 0.5 | 0 | 0 | 0.8 | 0 | 0.1 | 99.1 |
| OX1164 | 0 | 0.5 | 9.6 | 18.5 | 5.3 | 3.1 | 0 | 12.9 | 0 | 0 | 0 | 50 | 0 | 0 | 0 | 0 | 0 | 99.9 |
| T2218 | 2.8 | 2.3 | 23.3 | 29.8 | 11.5 | 8.5 | 0.2 | 18.7 | 1.2 | 0.6 | 0 | 1.1 | 0 | 0 | 0 | 0 | 0 | 100 |
| T2244 | 2 | 1.8 | 29.8 | 32.8 | 14.1 | 3.6 | 0.2 | 10.4 | 1.6 | 1.3 | 0.8 | 0.4 | 0 | 0 | 0.3 | 0 | 0 | 99.1 |
| Lee_1019 | 3.6 | 0.8 | 18.8 | 20.1 | 10.2 | 3.5 | 0.4 | 5.3 | 17.5 | 10.5 | 5.9 | 0.2 | 0 | 0 | 1.4 | 0 | 0.2 | 98.4 |
| OX1587 | 18.3 | 9.1 | 6 | 32.4 | 4.6 | 1.6 | 0.6 | 14.7 | 0.3 | 0 | 0 | 0 | 0 | 0 | 0 | 0 | 0 | 87.6 |
| A6691 | 15.2 | 13.4 | 10.5 | 11 | 30.4 | 2.1 | 0.2 | 1.1 | 3.1 | 3.1 | 0 | 3.1 | 0 | 0 | 3.1 | 3.1 | 0 | 99.4 |
| M4490 | 2.3 | 0.8 | 19.8 | 28 | 9.7 | 1.6 | 0.3 | 1.7 | 5.5 | 14.1 | 1.6 | 2 | 0 | 9.4 | 1.6 | 1.6 | 0 | 100 |
| A6939 | 3.4 | 7.3 | 15.2 | 27.8 | 16 | 5.3 | 0.3 | 22 | 1.3 | 0.8 | 0 | 0.4 | 0 | 0 | 0 | 0 | 0 | 99.8 |
| M3451 | 2.6 | 1.7 | 22.9 | 31 | 11.3 | 7.8 | 0.2 | 19.3 | 1.2 | 0.7 | 0 | 0.8 | 0 | 0 | 0.2 | 0.2 | 0 | 99.9 |
| T2222 | 0 | 0.6 | 39 | 39.7 | 16.7 | 0.4 | 0 | 3.6 | 0 | 0 | 0 | 0 | 0 | 0 | 0 | 0 | 0 | 100 |
| OX1545 | 16 | 0.8 | 18 | 19.9 | 9.4 | 4.6 | 0.4 | 4.3 | 6.7 | 3.7 | 6.3 | 3.2 | 0 | 0 | 3.1 | 3.1 | 0 | 99.5 |
| V901 | 0 | 0.6 | 41.4 | 40.1 | 15.3 | 0.4 | 0 | 2.1 | 0 | 0 | 0 | 0 | 0 | 0 | 0 | 0 | 0 | 99.9 |
| OX1716 | 2.9 | 5.2 | 20.3 | 30.1 | 16.3 | 4.3 | 0.5 | 16.3 | 1.1 | 0.8 | 0.8 | 0.6 | 0 | 0 | 0.4 | 0.4 | 0 | 100 |
| CH151 | 2.9 | 0.9 | 29.7 | 27 | 12.4 | 6.8 | 0.3 | 17.7 | 0 | 0 | 0 | 2.3 | 0 | 0 | 0 | 0 | 0 | 100 |
| Lee_1034 | 6.3 | 6.5 | 20.7 | 32.6 | 20.1 | 0.2 | 0 | 13.6 | 0 | 0 | 0 | 0 | 0 | 0 | 0 | 0 | 0 | 100 |
| A10055 | 11.5 | 0.4 | 34 | 29 | 13.9 | 5.4 | 2.3 | 1.1 | 0.8 | 0 | 0 | 1.6 | 0 | 0 | 0 | 0 | 0 | 100 |
| OX1599 | 5 | 2.7 | 21.4 | 23.3 | 12.5 | 3.9 | 0.9 | 9 | 9.2 | 5 | 4 | 0.6 | 0 | 0 | 1.1 | 1.1 | 0.2 | 99.9 |
| A2225 | 0 | 0.5 | 9.6 | 18.5 | 5.3 | 3.1 | 0 | 12.9 | 0 | 0 | 0 | 50 | 0 | 0 | 0 | 0 | 0 | 99.9 |
| OX1478 | 0.9 | 1.5 | 21.7 | 34.5 | 11 | 6.1 | 0 | 21.7 | 0.6 | 0.5 | 0 | 0.5 | 0 | 0 | 0.5 | 0.5 | 0 | 100 |
| M2146 | 0 | 0 | 0 | 100 | 0 | 0 | 0 | 0 | 0 | 0 | 0 | 0 | 0 | 0 | 0 | 0 | 0 | 100 |
| A7175 | 4.7 | 1.5 | 27.6 | 28.1 | 14.4 | 3.2 | 0.5 | 3.1 | 0 | 3.1 | 0 | 3.9 | 0 | 0 | 3.1 | 3.1 | 0 | 96.3 |
| A9386 | 4.1 | 0.4 | 36 | 33.9 | 14.8 | 3.2 | 0.7 | 4.6 | 0.6 | 0 | 0 | 1.8 | 0 | 0 | 0 | 0 | 0 | 100.1 |
| A6169 | 2.9 | 3.7 | 16.2 | 27.2 | 10.2 | 9.9 | 0.2 | 23.6 | 3.2 | 2.7 | 0 | 0 | 0 | 0 | 0 | 0 | 0 | 99.8 |
| OX1668 | 3.7 | 1.9 | 23.2 | 28.4 | 11.7 | 5.3 | 0.5 | 14 | 2.7 | 2.3 | 3.1 | 0.7 | 0 | 0 | 1.1 | 1.1 | 0.2 | 99.9 |
| A6987 | 4.7 | 0.8 | 28.8 | 30.6 | 12.4 | 5.3 | 0.5 | 9.8 | 2.4 | 1.9 | 0 | 1.7 | 0 | 0 | 0.4 | 0.4 | 0 | 99.7 |
| A8671 | 27.1 | 0.2 | 15.9 | 42.6 | 7.4 | 0.8 | 0.3 | 1.3 | 2.2 | 2 | 0 | 0 | 0 | 0 | 0 | 0 | 0 | 99.8 |
| OX1639 | 4.6 | 2.9 | 20.6 | 23.1 | 12.2 | 4 | 0.6 | 9.2 | 7.8 | 5.7 | 4.2 | 1.4 | 0 | 0 | 1.7 | 1.7 | 0.2 | 99.9 |
| OX1573 | 5 | 2.7 | 21.4 | 23.3 | 12.5 | 3.9 | 0.9 | 9 | 9.2 | 5 | 4 | 0.6 | 0 | 0 | 1.1 | 0 | 0.2 | 98.8 |
| F89230 | 2.1 | 2.1 | 21 | 30 | 11.3 | 7.8 | 0.1 | 20.1 | 1.7 | 1.1 | 0.8 | 0.7 | 0 | 0 | 0.5 | 0.5 | 0 | 99.8 |
| A9945 | 0.9 | 1.5 | 21.7 | 34.5 | 11 | 6.1 | 0 | 21.7 | 0.6 | 0.5 | 0 | 0.5 | 0 | 0 | 0.5 | 0.5 | 0 | 100 |
| A10040 | 1.5 | 1.4 | 10.8 | 63.8 | 6.2 | 2.9 | 0.1 | 7.1 | 1.9 | 1.6 | 1.6 | 0.2 | 0 | 0 | 0.4 | 0.4 | 0.1 | 100 |
| A9785 | 8.1 | 1.7 | 27.4 | 26.5 | 12.8 | 5.1 | 1.5 | 6.2 | 2.5 | 1.9 | 3.1 | 1 | 0 | 0 | 1 | 1 | 0.2 | 100 |
| OX1586 | 3.3 | 3.9 | 24.6 | 24 | 11.1 | 3.9 | 0.5 | 5.4 | 2.9 | 2.3 | 0.4 | 2 | 0 | 12.5 | 1.6 | 1.6 | 0 | 100 |
| A8792 | 2.6 | 0.9 | 25.6 | 32.7 | 11.7 | 5.9 | 0.2 | 16.7 | 1.2 | 0.9 | 0 | 0.9 | 0 | 0 | 0.2 | 0.2 | 0 | 99.7 |
| A9903 | 4.3 | 1 | 25.4 | 31.5 | 11.4 | 4.7 | 0.7 | 11.1 | 2.1 | 1.8 | 0 | 2.3 | 0 | 0 | 1.8 | 1.8 | 0 | 99.9 |
| A2945 | 29.9 | 7 | 10.8 | 9.2 | 5.8 | 1.6 | 0.2 | 2.6 | 15.4 | 8.5 | 5.9 | 0 | 0 | 0 | 1.4 | 1.4 | 0.2 | 99.9 |
| A8640 | 50 | 0.5 | 10.2 | 18 | 5.4 | 3.1 | 0 | 12.8 | 0 | 0 | 0 | 0 | 0 | 0 | 0 | 0 | 0 | 100 |
| A10138 | 3.4 | 1.8 | 23.2 | 31.7 | 10.9 | 6.9 | 0.3 | 17.2 | 1 | 0.9 | 0 | 1.5 | 0 | 0 | 0.5 | 0.5 | 0 | 99.8 |
| A10746 | 0 | 0.6 | 41.4 | 40.1 | 15.3 | 0.4 | 0 | 2.1 | 0 | 0 | 0 | 0 | 0 | 0 | 0 | 0 | 0 | 99.9 |
| A6390 | 1.1 | 1.4 | 32.4 | 34.8 | 13.4 | 4.7 | 0 | 11.5 | 0.3 | 0 | 0 | 0.4 | 0 | 0 | 0 | 0 | 0 | 100 |
| A10670 | 0 | 0.6 | 41.4 | 40.1 | 15.3 | 0.4 | 0 | 2.1 | 0 | 0 | 0 | 0 | 0 | 0 | 0 | 0 | 0 | 99.9 |
| Lee_1356 | 2.6 | 2.1 | 20.1 | 31 | 10.8 | 7.9 | 0.2 | 20 | 2.6 | 1.6 | 0 | 0.5 | 0 | 0 | 0.2 | 0.2 | 0 | 99.8 |
| A6255 | 0 | 0 | 0 | 0 | 0 | 0 | 0 | 0 | 0 | 0 | 0 | 50 | 50 | 0 | 0 | 0 | 0 | 100 |
| OX1370 | 11.5 | 0.4 | 34 | 29 | 13.9 | 5.4 | 2.3 | 1.1 | 0.8 | 0 | 0 | 1.6 | 0 | 0 | 0 | 0 | 0 | 100 |
| OX1460 | 8.1 | 1.7 | 27.4 | 26.5 | 12.8 | 5.1 | 1.5 | 6.2 | 2.5 | 1.9 | 3.1 | 1 | 0 | 0 | 1 | 1 | 0.2 | 100 |
| A11294 | 50 | 0 | 0 | 0 | 0 | 0 | 0 | 0 | 0 | 0 | 0 | 25 | 25 | 0 | 0 | 0 | 0 | 100 |
| A8591* | 0 | 0 | 0 | 25 | 0 | 0 | 0 | 0 | 0 | 0 | 0 | 25 | 0 | 0 | 0 | 0 | 0 | 50 |
| OX1701* | 0.4 | 1 | 30.8 | 32.8 | 14.1 | 4.2 | 0 | 16.6 | 0 | 0 | 0 | 0 | 0 | 0 | 0 | 0 | 0 | 99.9 |
| A6535* | 34.8 | 7.2 | 11.5 | 16.3 | 6.9 | 6 | 0.4 | 9.7 | 2.9 | 2.9 | 0 | 0 | 0 | 0 | 0.2 | 0.2 | 0 | 99 |
| OX1499* | 29.3 | 1 | 15.9 | 21.7 | 9.2 | 5.3 | 0.4 | 7.9 | 4.4 | 4.3 | 0 | 0 | 0 | 0 | 0 | 0 | 0 | 99.4 |
| A9302* | 2.4 | 0.8 | 19.1 | 28.4 | 9.2 | 1.9 | 0.3 | 1.6 | 2.5 | 18.8 | 0 | 2.1 | 0 | 9.4 | 1.8 | 1.8 | 0 | 100.1 |
| A9333* | 31.9 | 14.7 | 7.5 | 8.2 | 3.3 | 1.7 | 0.2 | 2.7 | 0.7 | 14.7 | 0 | 0.1 | 1.6 | 6.3 | 0 | 0 | 0 | 93.6 |
| A7524* | 3.6 | 1.4 | 24.5 | 29.8 | 11.8 | 7.1 | 0.3 | 15.5 | 2.2 | 1.5 | 0 | 1.1 | 0 | 0 | 0.4 | 0.4 | 0 | 99.6 |
| Phal.Equestris* | 100 | 0 | 0 | 0 | 0 | 0 | 0 | 0 | 0 | 0 | 0 | 0 | 0 | 0 | 0 | 0 | 0 | 100 |
| OX1408* | 2.3 | 1.4 | 23.5 | 34.1 | 11.5 | 5.5 | 0.2 | 19 | 1.1 | 0.8 | 0 | 0.4 | 0 | 0 | 0.1 | 0.1 | 0 | 100 |
| A5724* | 2 | 0 | 15.1 | 13.7 | 5.5 | 0.4 | 0 | 0.9 | 0 | 31.3 | 0 | 0 | 6.3 | 25 | 0 | 0 | 0 | 100.2 |
| OX1233* | 0 | 0.6 | 41.4 | 40.1 | 15.3 | 0.4 | 0 | 2.1 | 0 | 0 | 0 | 0 | 0 | 0 | 0 | 0 | 0 | 99.9 |
| A7403* | 22.6 | 3.8 | 13.1 | 12.6 | 7.1 | 0 | 0.2 | 2.8 | 15 | 9.8 | 7.4 | 0 | 0 | 0 | 0 | 0 | 0 | 94.4 |
| Ratio(%) | 10 | 3 | 20 | 30 | 11 | 4 | 0 | 9 | 3 | 3 | 1 | 3 | 1 | 2 | 1 | 1 | 0 |  |
| N1414 | 50.4 | 18.8 | 11.8 | 10 | 4.6 | 2.7 | 1.4 | 0.4 | 0 | 0 | 0 | 0 | 0 | 0 | 0 | 0 | 0 | 100.1 |
| *indicates unique or distinct ancestors in the pedigrees of the core collection. | | | | | | | | | | | | | | | | | | |
